# A meta-learning framework to mitigate negative transfer in transfer learning applicable to drug design

**DOI:** 10.1038/s41598-025-22058-3

**Published:** 2025-10-09

**Authors:** Antonia Mera, Martin Vogt, Jürgen Bajorath

**Affiliations:** 1 Department of Life Science Informatics and Data Science, LIMES Program Unit Chemical Biology and Medicinal Chemistry, B-IT, Friedrich-Hirzebruch-Allee 5/6, Bonn, Germany; 2 Lamarr Institute for Machine Learning and Artificial Intelligence, University of Bonn, Friedrich-Hirzebruch-Allee 5/6, D-53115 Bonn, Germany

**Keywords:** Deep learning, Low-data regimes, Transfer learning, Meta-learning, Adaptive knowledge transfer, Model generalization, Negative transfer, Cheminformatics, Computational biology and bioinformatics, Mathematics and computing

## Abstract

Data sparseness is a major limiting factor for deep machine learning. In the natural sciences, data distributions are heterogeneous. For instance, in chemistry and early-phase drug discovery, compound and molecular property data are typically sparse compared to data in other fields such as particle physics or genome biology. For machine learning in low-data regimes, approaches such as transfer learning or meta-learning have been introduced. These learning strategies are conceptually related but algorithmically distinct and typically applied independently. They share the common goal of facilitating knowledge transfer between domains with related prediction tasks and varying data availability. We were interested in combining meta- and transfer learning into a coherent framework, primarily for deep learning in cheminformatics. Therefore, we introduce a new meta-learning algorithm designed to complement transfer learning. It identifies an optimal subset of training instances and determines weight initializations for deriving base models that can then be fine-tuned under conditions of data scarcity. Given its ability to identify preferred training samples, the meta-learning algorithm balances negative transfer between source and target domains, which represents a major caveat for transfer learning. In an extensive proof-of-concept application, inhibitors of protein kinases were predicted following data reduction using combined meta- and transfer learning, revealing statistically significant increases in model performance and effective control of negative transfer.

**Supplementary Information:**

The online version contains supplementary material available at 10.1038/s41598-025-22058-3.

## Introduction

 In early-phase drug discovery and design, compound and molecular property data are typically sparse, which often limits meaningful deep machine learning applications. For related prediction tasks, data constraints can be circumvented through the application of methods such as transfer learning, which aims to learn features that are transferable between tasks to compensate for sparse data^1^. Transfer learning formally distinguishes between the source domain consisting of one or more tasks that are related to the target domain representing the primary task(s) of interest. For instance, an exemplary transfer learning strategy involves pre-training of a model for a data-restricted task (target domain) on data for a related task (source domain), followed by fine-tuning on the data-restricted tasks^1^.

Sequential single-task learning can be further extended using multi-task transfer learning models. While multi-task learning is also applicable without transfer components, it represents a suitable framework for transfer learning. For example, Ye et al.^2^ used a bioactivity data set comprising ligands of 157 target proteins to pre-train a multi-task neural network composed of a feature extractor and a task layer. Weights from pre-training were then transferred to another multi-task neural network derived to predict pharmacokinetic parameters for a set of approved drugs. This transfer learning strategy reached higher prediction accuracy than conventional machine learning models and had better generalization ability^2^. While transfer learning is a method of choice for predictions in low-data regimes, it is generally difficult to confidently select tasks where transfer learning will be superior to other machine learning approaches. For example, although compound activity prediction for members of the same protein family basically represents a suitable task for transfer learning, optimal learning conditions for such predictions might substantially differ^3^. Therefore, methods are desirable to quantify task similarity and guide the selection of the source domain. For instance, similarity between target and potential source tasks can be assessed based on latent data representations learned by graph neural networks individually pre-trained for each task^4^. This approach is primarily applicable to uniform data such as compounds having a specific activity. In addition, similarity (or distance) scoring is also applicable by combining similarity assessment for data representations such as protein sequence and chemical space embeddings^5^. However, deriving a task similarity measure for multiple tasks or combinations of tasks typically is computationally demanding, emphasizing the need for an adaptive weighting algorithm. Meta-learning is another approach for addressing prediction tasks in low-data regimes^6^. Different from the standard pre-training and fine-tuning scheme of transfer learning encompassing different domains, meta-learning aims to derive models that can effectively adapt to new low-data tasks, without the need for extensive additional training. In a typical meta-learning framework, a base model is trained in an inner loop to address an individual task, while an outer loop optimizes a given “meta-objective” such as learning a weight initialization to improve generalization across tasks^6^. The ability of meta-learning to meet this objective for limited training instances also depends on task similarity^7^.

For neural network architectures, weight distributions for modeling of related tasks can be derived in different ways. For example, the Meta-Weight-Net algorithm was designed to learn sample weights based on their classification loss^8^. Therefore, a shallow neural network uses the loss from a base model prediction of a test instance as input and derives a weight for this instance. Utilizing the loss in a weighting scheme can guide learning based on the hardness of each instance to be classified by the model, as implemented in the AdaBoost algorithms^9^. However, a larger feature space, as can be covered by the new algorithm introduced herein, is likely to contain increasing amounts of important information, especially when addressing multiple tasks, which substantially aids in deriving a weighting scheme. Furthermore, the Model-Agnostic Meta‐Learning (MAML) algorithm searches for weight initializations that only require a few gradient steps to train a base model^10^. While Meta-Weight-Net and related algorithms derive weights for individual samples, MAML operates on tasks that are expected to support a given target task. For example, MAML has been applied to increase the learning effectiveness and performance of transformer-based chemical language models in predicting potent compounds based on weakly active templates^11^. However, the MAML approach cannot be applied if source and target tasks lack significant similarity^12,13^, leading to so-called negative transfer^14^, which is known to compromise transfer learning. Negative transfer of task information decreases the performance of a transfer learning model relative to the base model^14^. In contrast to MAML, our meta-learning method uses both sample and task information for its unique meta-objective to mitigate negative transfer, which offers opportunities in complementing methods designed to use information from multiple related tasks. Importantly, negative transfer can also take place at the instance level, for example, due to the presence of activity or selectivity cliffs in compound data sets, which the currently available meta-learning frameworks including MAML do not take into account. Moreover, although techniques to mitigate negative transfer have been introduced^15,16^, there currently is no method available that regulates negative transfer with meta-learning. This provides an opportunity for combining meta- and transfer learning. However, although transfer learning and meta-learning are conceptually related, potential synergies between these approaches for machine learning in low-data regimes have thus far not been explored.

To combine the strengths of transfer and meta-learning,  our new meta-learning algorithm specifically complements standard transfer learning. The algorithm combines task and sample information and its unique meta-objective is the optimization of the generalization potential of a pre-trained transfer learning model in the target domain. Therefore, a model is pre-trained in a source domain using weights determined by the meta-learning algorithm. This provides a basis for effective fine-tuning of the transfer learning model in a target domain. The unique feature of the meta-learning algorithm enabling this combined approach is the identification of an optimal subset of source samples for pre-training of the transfer learning model. The ability to optimize training sample selection makes it possible to algorithmically balance negative transfer between the source and target domains. This major limitation of transfer learning is directly addressed for the first time by combining meta- and transfer learning with our new algorithm.

## Methods

### Compounds, activity data, and molecular representations

For our analysis, a protein kinase inhibitor (PKI) data set was generated. Therefore, protein kinases (PKs) comprising the human kinome^17^ and activity data for PKIs were systematically collected from ChEMBL^18^ (version 34) and BindingDB^19^ and combined according to Vossen et al.^20^, yielding more than 450,000 PKIs with activity against 461 PKs. The PKI data set was filtered to only contain (assay-independent) K_i_ values as activity annotations and compounds with a molecular mass of less than 1000 Da. Structures of qualifying compounds were standardized and canonical nonisomeric SMILES stings^21^ were generated using RDKit^22^. For multiple available K_i_ values per compound $\:c$ for a given PK, the geometric mean was calculated if these values met the condition $\:\frac{K{i}_{max}^{c}}{K{i}_{min}^{c}}\le\:10$ (if not, the measurements were discarded). The final curated data set contained 7098 unique PKIs with activity against 162 PKs and a total of 55,141 PK annotations.

For activity-based classification, K_i_ values were transformed into a binary (active/inactive) format by applying a potency threshold of 1000 nM. Accordingly, compounds with a K_i_ value below or above this threshold were labeled as active and inactive, respectively. Of note, the choice of this threshold was motivated by practical medicinal chemistry where PKIs with 1000 nM activity are typically considered inactive, given the need for at least low-nanomolar PKIs for drug development.

For transfer and meta-learning, 19 PKs with at least qualifying 400 PKIs and 25–50% of these PKIs classified as active were selected. Table 1 summarizes these data sets. The total number of PKIs and the number of classified actives per set ranged from 474 to 1028 and 151–363, respectively.

As a molecular representation for machine learning, the extended connectivity fingerprint^23^ with bond diameter of 4 (ECFP4) and a constant size of 4096 bits was generated from SMILES strings of compounds using RDKit.

**Table 1:** Protein kinase inhibitor sets.

Abbreviation	Protein kinase	# PKIs	# Actives
	AGC Ser/Thr protein kinase family		
PKN2	Serine/threonine-protein kinase N2	699	183
PRKX	cAMP-dependent protein kinase catalytic subunit PRKX	733	197
	Tyr protein kinase family		
BLK	Tyrosine-protein kinase Blk	635	216
	CAMK Ser/Thr protein kinase family		
DAPK3	Death-associated protein kinase 3	892	239
MELK	Maternal embryonic leucine zipper kinase	564	171
MKNK2	MAP kinase-interacting serine/threonine-protein kinase 2	665	195
STK17A	Serine/threonine-protein kinase 17 A	576	186
	CMGC Ser/Thr protein kinase family		
CDK8	Cyclin-dependent kinase 8	474	195
CLK2	Dual specificity protein kinase CLK2	855	231
DYRK1A	Dual specificity tyrosine-phosphorylation-regulated kinase 1 A	1028	336
HIPK2	Homeodomain-interacting protein kinase 2	669	204
HIPK4	Homeodomain-interacting protein kinase 4	599	151
	STE Ser/Thr protein kinase family		
MAP4K2	Mitogen-activated protein kinase kinase kinase kinase 2	858	245
MAP4K4	Mitogen-activated protein kinase kinase kinase kinase 4	971	363
MAP4K5	Mitogen-activated protein kinase kinase kinase kinase 5	666	238
MINK1	Misshapen-like kinase 1	644	165
SLK	STE20-like serine/threonine-protein kinase	677	221
STK3	Serine/threonine-protein kinase 3	796	204
	TKL Ser/Thr protein kinase family		
LIMK1	LIM domain kinase 1	850	227

## Method formulation

Standard transfer learning leverages knowledge from a source domain with abundant data to improve learning in a low-data target domain. As discussed above, performance in the target domain can be compromised if the two data domains are not sufficiently similar, causing negative transfer^14^.

**Fig. 1:**
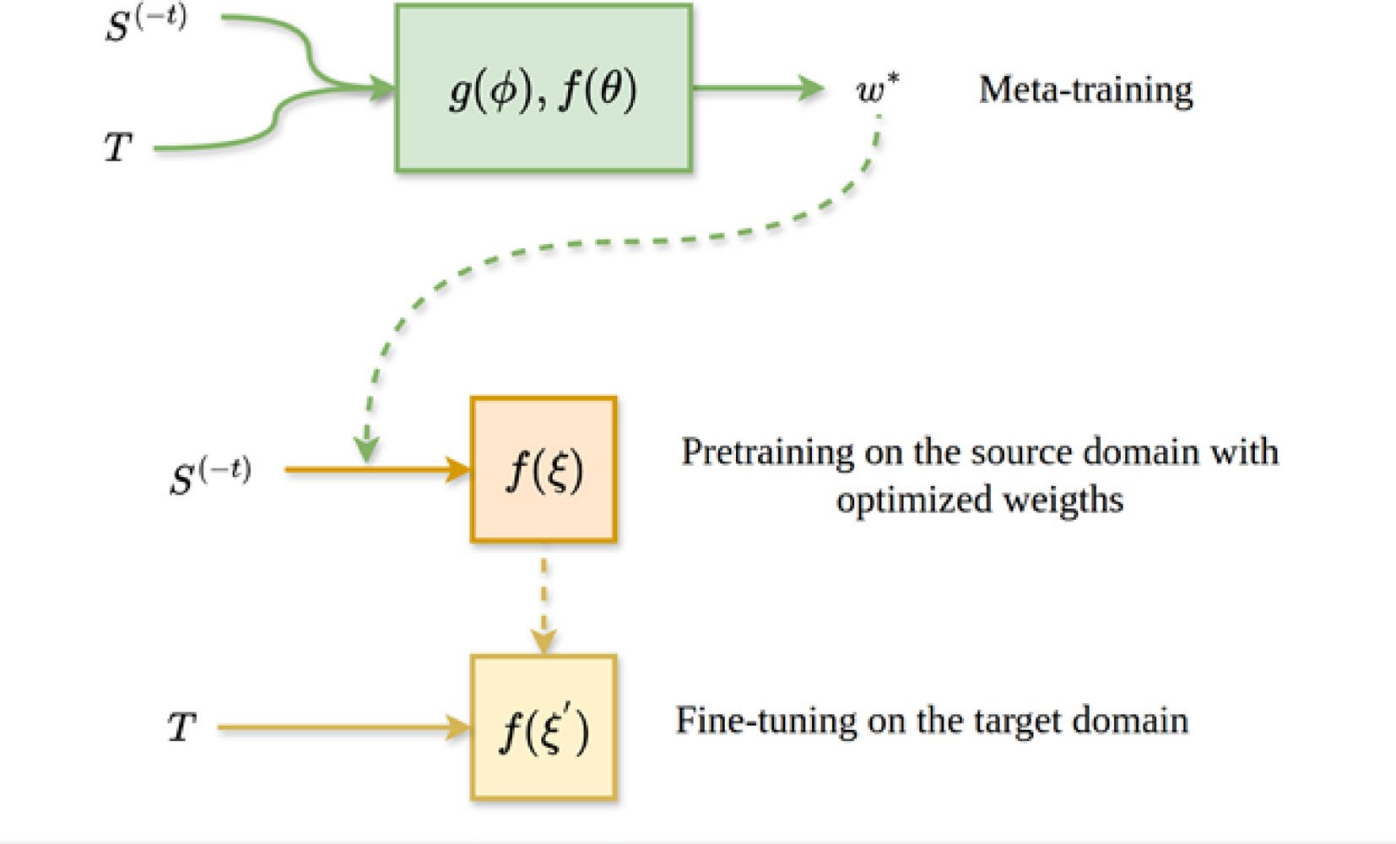
Meta-learning principles. The meta-training procedure uses a base model $\:f$ and meta- model $\:g$ to optimize source $\:{S}^{\left(-t\right)}$ sample weights $\:{w}^{}$. Parameters of the base model $\:f$ are re-initialized and adjusted using the weighting scheme learned by the meta-model. Fine-tuning is then applied to the target domain $\:T$.

The meta-learning algorithm introduced herein aims to mitigate negative transfer. Therefore, a meta-model must derive weights for the source data points adjusting the relative contributions of samples during pre-training of a base model (Fig. 1). We apply the method to PKI data sets since compound information for multiple PKs can be readily transferred to a PK with reduced compound data, as detailed below.

First, two data sets are specified including a target data set (inhibitors of a data-reduced PK): $\:{T}^{\left(t\right)}=\left\{\left({x}_{i}^{t},{y}_{i}^{t},{s}^{t}\right)\right\}$

and a source data set (containing PKIs of multiple PKs excluding the target PK):

$$\:{S}^{\left(-t\right)}={\left\{\left({x}_{j}^{k},{y}_{j}^{k},{s}^{k}\right)\right\}}_{k\ne\:t}$$

Here, $\:x$ represents the molecule, $\:y$ is the label, and $\:s$ is a protein sequence representation.

Next, we define the two models for the meta-learning framework as follows:

The base model $\:f$ with parameters $\:\theta\:$ for classifying active vs. inactive compounds is trained on the source data $\:{S}^{\left(-t\right)}$ with a weighted loss function, in which the weights correspond to the weight predictions of a meta-model $\:g$ for each data point. For the target data set $\:T$, the base model predicts the binary activity states of the compounds in the target training data set. From the predicted activity states, the validation loss is calculated, adding a second layer of optimization using the validation loss to update the meta-model.

The meta-model $\:g$ with parameter $\:\varphi\:$ predicts weights for training data of the base model. For a source data set $\:{S}^{\left(-t\right)}$ composed of $\:K$ PKs together with their ligands $\:{\left\{\left({x}_{j}^{k},{y}_{j}^{k},{s}^{k}\right)\right\}}_{k\ne\:t}$, the meta-model assesses how informative the $\:{j}^{th}$ compound of the $\:{k}^{th}$ PK is for the target PK $\:t$ by predicting a weight $\:{w}_{j}^{k}$. Weight predictions are limited to the interval $\:\left(\text{0,1}\right)$ by the sigmoid function to avoid extreme predictions during training.

The weights predicted by the meta-model are then used to train the base model with the following weighted binary cross entropy

1 $$\:{L}_{train}=\frac{{\sum\:}_{k=1,k\ne\:t}^{K}{\sum\:}_{j=1}^{{N}_{k}}g\left({x}_{j}^{k},{s}^{k};\varphi\:\right)\text{BCE}\left({y}_{j}^{k},f\left({x}_{j}^{k};\theta\:\right)\right)}{{\sum\:}_{k=1,k\ne\:t}^{K}{\sum\:}_{j=1}^{{N}_{k}}g\left({x}_{j}^{k}, {s}^{k};\varphi\:\right)}$$

where $$\:\text{BCE}\left(u,\stackrel{\prime }{u}\right)=u\text{log}\left(\stackrel{\prime }{u}\right)+\left(1-u\right)\text{log}\left(1-\stackrel{\prime }{u}\right)$$.

The central idea of the method is optimizing $\:{w}_{j}^{k}$ values and training the base model on the source data $\:{S}^{\left(-t\right)}$ according to Eq. 1. Accordingly, the base model should improve performance on the target data $\:T$ compared to its counterpart model that is trained with the unweighted loss. For weight optimization, the validation loss of the base model is estimated based on the target training data $\:T$ and used to train the meta-model. The loss function for the validation loss is the standard binary cross-entropy (BCE) loss and computed as:

2 $$\:{L}_{val}=\frac{{\sum\:}_{i=1}^{{N}_{t}}\text{BCE}\left({y}_{i}^{t},f\left({x}_{i}^{t};\theta\:\right)\right)}{N}$$

Training of the meta-model is based on these two losses. First, the validation loss with respect to parameter $\:\varphi\:$ needs to be computed. Since $\:{L}_{val}$ is only a function of $\:\theta\:$, the indirect dependence of $\:\varphi\:$ on $\:\theta\:$ is added and the chain rule is applied. The meta-gradients are of the general form:

3 $$\:\frac{\partial\:{L}_{\text{val}}}{\partial\:\varphi\:}=\frac{\partial\:{L}_{\text{val}}}{\partial\:\theta\:}\cdot\:\frac{\partial\:\theta\:}{\partial\:\varphi\:}$$

where $\:\partial\:\theta\:$ can be derived by differentiating the backpropagation of the base model with respect to the meta-model’s $\:\varphi\:$ parameters.

4 $$\:\partial\:\theta\:=-{\eta\:}_{\theta\:}\cdot\:\frac{\partial\:{L}_{\text{train}}}{\partial\:\theta\:}\Rightarrow\:\frac{\partial\:\theta\:}{\partial\:\varphi\:}=-{\eta\:}_{\theta\:}\cdot\:\frac{{\partial\:}^{2}{L}_{\text{train}}}{\partial\:\theta\:\partial\:\varphi\:}$$

Combining Eqs. 3 and 4 yields:

5 $$\:\frac{\partial\:{L}_{\text{val}}}{\partial\:\varphi\:}=\frac{\partial\:{L}_{\text{val}}}{\partial\:\theta\:}\cdot\:\left(-{\eta\:}_{\theta\:}\cdot\:\frac{{\partial\:}^{2}{L}_{\text{train}}}{\partial\:\theta\:\partial\:\varphi\:}\right)$$

**Fig. 2:**
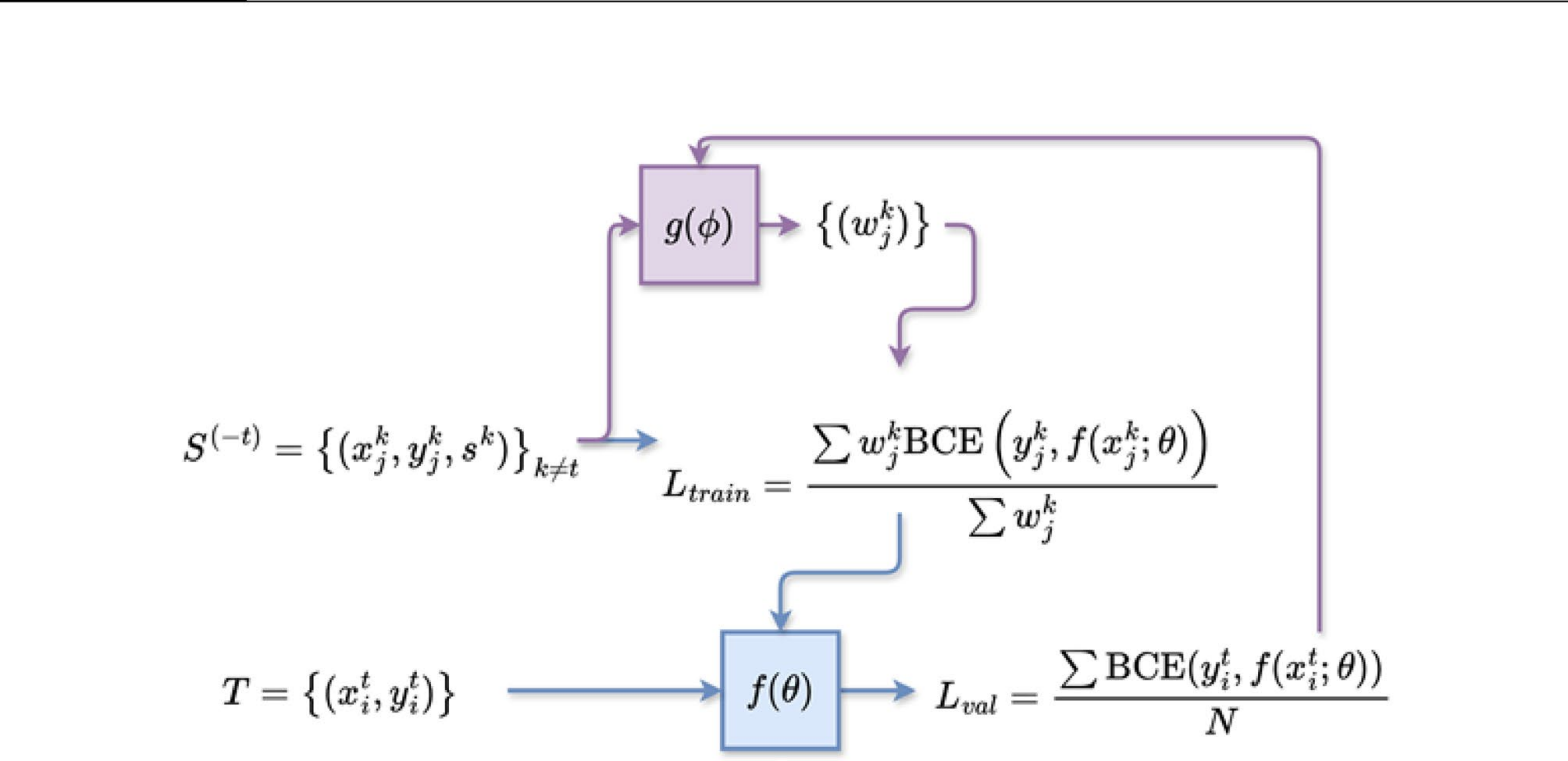
Meta-learning framework. The base model f is trained using the source data $\:S$ with the weighted loss $\:{L}_{train}$ and validated using the training target data set $\:T$ with the loss function $\:{L}_{val}$. The validation loss is used for updating the meta-model g and optimizing the predicted weights.

The backpropagation on the meta-model is then formulated as:

6 $$\:\partial\:\varphi\:=-{\eta\:}_{\varphi\:}\left(-{\eta\:}_{\theta\:}\frac{\partial\:{L}_{\text{val}}}{\partial\:\theta\:}\frac{{\partial\:}^{2}{L}_{\text{train}}}{\partial\:\theta\:\partial\:\varphi\:}\right)=+{\stackrel{\prime }{\eta\:}}_{\varphi\:}\frac{\partial\:{L}_{\text{val}}}{\partial\:\theta\:}\frac{{\partial\:}^{2}{L}_{\text{train}}}{\partial\:\theta\:\partial\:\varphi\:}$$

with learning rates $\:{\eta\:}_{\varphi\:}$ and $\:{\eta\:}_{\theta\:}$ that are combined to $\:{\stackrel{\prime }{\eta\:}}_{\varphi\:}={\eta\:}_{\varphi\:}\cdot\:{\eta\:}_{\theta\:}$. For practical purposes, $\:{\stackrel{\prime }{\eta\:}}_{\varphi\:}$ is treated as an independent parameter. Algorithm 1 summarizes the meta-training procedure and Fig. 2 illustrates the meta-learning framework.

**Figure:**
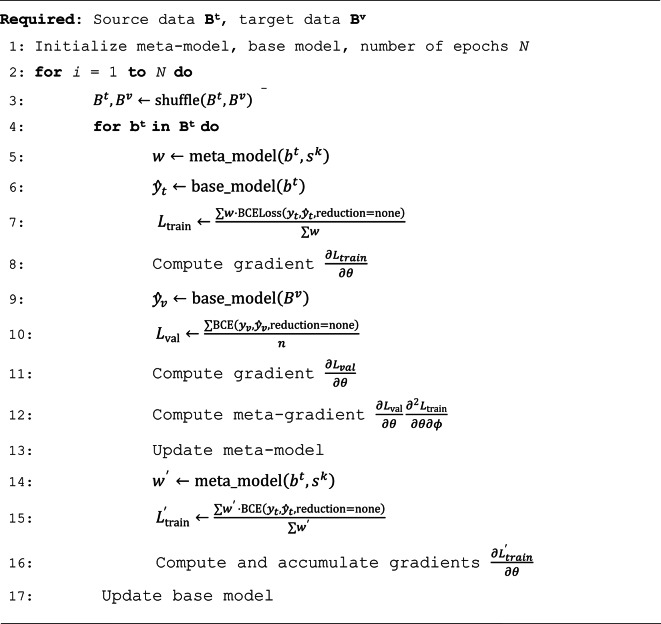
Algorithm 1. Meta-training.

### Noisy meta-gradients

Numerical second-order gradients have high variance, which can destabilize meta-training and hinder its convergence. For preventing limited convergence, first-order approximations of the meta-gradients are often generated, which tends to reduce the accuracy of model updates^10^. We address unstable meta-training by adjusting the size of mini-batches, representing a factor known to influence the variance of gradients^24^. For small target data sets, training can take place in a single batch. For large source data sets, memory capacity is a serious constraint. Therefore, a different approach is applied by accumulating gradients through batches and updating the model only at the end of an epoch (Algorithm 1), thus effectively eliminating batch size as a significant hyperparameter. This algorithmic modification reduces the overall noise of the first-order gradients used to update the base model. Given the dependency of the meta-gradients on the backpropagation of the base model (Eq. 5), meta-training fluctuations are then stabilized.

### Monte Carlo dropout for preventing overfitting

In addition to noisy meta-gradients, overfitting is a common problem in heavily parameterized deep neural networks trained on scarce data. A widely used method for controlling overfitting is so-called dropout, that is, neurons are randomly deactivated during training with a pre-defined probability^25^. Monte Carlo dropout refers to neuron deactivation enabled during testing and is used to produce stochastic predictions for uncertainty estimates. It was shown to approximate Bayesian inference at low computational cost^26^ and further increase the predictive performance of a deep neural network compared to standard dropout^27^.

Monte Carlo dropout is incorporated into the meta-model neural network to mitigate overfitting. Therefore, during the pre-training phase of a base model, weights are sampled from the meta-model and used as a part of the weighted loss function (Eq. 1). The number of samples drawn for each compound in the source data set is equal to the number of pre-training epochs. The process of sampling creates a distribution of weights throughout the training phase. The standard deviation of this distribution is directly influenced by the dropout value^28^. High values can lead to weighted predictions that vary significantly between different epochs, hence complicating the learning process. The dropout rate was selected through grid search optimization and set to 0.2. Higher dropout rates led to a reduction in model performance (Supplementary Fig. 1). The process of training with the weighted loss is summarized in Algorithm 2.

**Figure:**
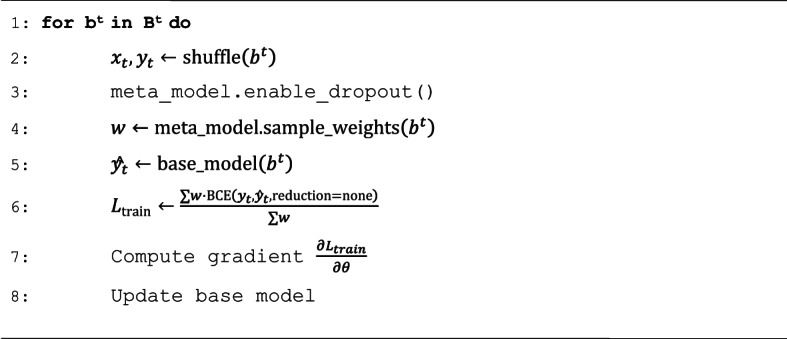
Algorithm 2. Pre-training with Monte Carlo dropout.

### Analysis set-up

To evaluate the efficiency of our meta-learning algorithm in preventing negative transfer, we compare the method with standard transfer learning. In both cases, a base model with the same architecture is pre-trained on the source data set $\:{S}^{\left(-t\right)}$ and fine-tuned on the target data set $\:T$ using the same fine-tuning protocol. However, the loss functions applied during pre-training with the source data differ. For meta-learning, the base model is trained using weighted BCE loss (with weights predicted by the meta-model) whereas standard transfer learning utilizes unweighted BCE loss for training.

The performance of the models is evaluated using two calculation settings. In the first setting, the compound spaces of the source and target domains overlap, that is, they contain compounds that have K_i_ measurements for target PK $\:T$ as well as for PKs in the source data set $\:{S}^{\left(-t\right)}$. In the second setting, compounds shared by the two domains are removed from the source data set $\:{S}^{\left(-t\right)}$ resulting in non-overlapping compound sets:

7 $$\:{S}^{\left(-t,-\cap\:\right)}=\left\{\left({x}^{k},{y}^{k},{s}^{k}\right)\in\:S|k\ne\:t\text{ and }{x}^{k}\notin\:{X}^{T}\right\}$$

Removal of the shared compounds increases the chemical distance between the source and target domain, resulting in increasing challenges for standard transfer learning, which are addressed by varying hyperparameter settings, as described in the next section.

To study differences in the information transfer from the source to the target domain, random forest (RF) models are derived as an additional control using scikit-learn^29^ with default parameter settings. In contrast to standard transfer and meta-learning, RF models are exclusively trained on the target domain. Notably, feedforward neural networks were also evaluated as a control, but had lower performance compared to RF. The performance of standard transfer and meta-learning relative to the neural network control model is reported in Supplementary Fig. 2.

### Model architectures and hyperparameters

**Fig. 3:**
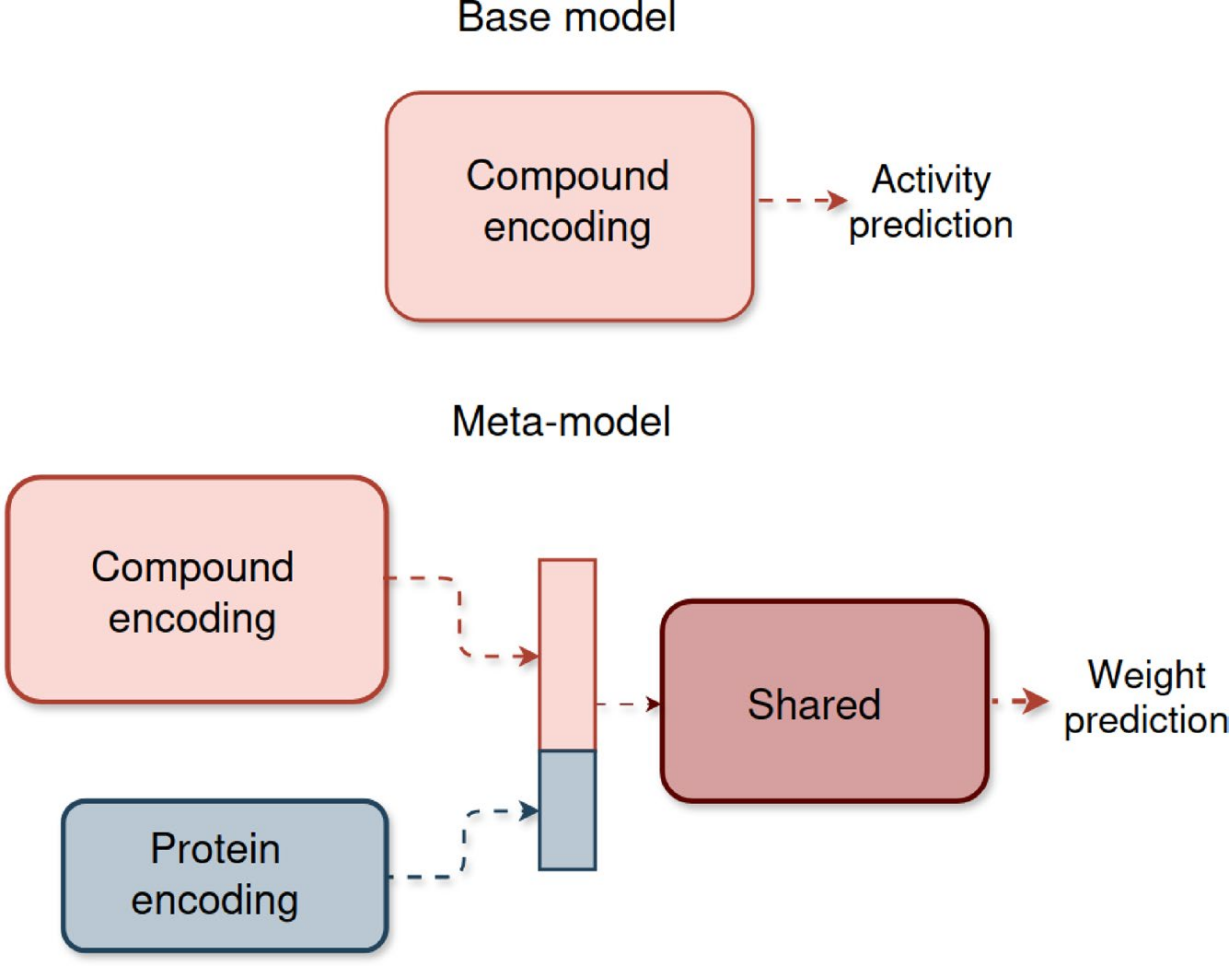
Model architectures. Shown is a schematic representation of the base model and meta-model. The base model is a feedforward neural network that takes a compound fingerprint as input and predicts the class label of this compound. The meta-model is a modular neural network that takes a compound fingerprint and a one-hot encoded representation of the target protein as input and predicts a weight for this compound-protein pair.

The base model is a feedforward neural network taking ECFP4 representations of compounds as input and producing binary class label predictions (active vs. inactive) as output (Fig. 3). The architecture of the meta-model is slightly more complex, modular, and depends on two types of input data including (i) ECFP4 of a compound (PKI) and (ii) a one-hot encoded representation of the sequence of the associated protein (PK). The modular meta-model network processes these two inputs independently in parallel before the latent representations are concatenated and further processed in additional layers. The sigmoid activation function is applied to the output of the last layer resulting in a predicted weight for the PKI-PK input pair (Fig. 3).

The first two layers of the base model form a so-called bottleneck where the input is projected to a lower-dimensional space and then back to the original dimensionality. This architectural feature enables the construction of a representation containing features characteristic of PKs for learning of PK-dependent data patterns. To retain the learned patterns after pre-training, the bottleneck feedforward layers and subsequent normalization layers^30^ are frozen, while the following layers are subjected to fine-tuning. This strategy was found to be beneficial for transfer learning using small data sets^31^.

For base model and meta-model training, a learning rate of $\:{10}^{-5}$ is applied in combination with the Adam optimizer^32^. The batch size of the source data set ranges from 512 to 4096, depending on the data set size (and GPU memory constraints). The number of meta- and pre-training epochs is set to 200 and 150, respectively, for the first calculation setting (see above) and to 100 and 100, respectively, for the second setting. In both cases, fine-tuning is carried out over 100 epochs. The base model and meta-model are implemented with PyTorch^33^. Notably, data samples with initially assigned weights close to zero are best excluded from pre-training since they do not significantly contribute to the loss. Frequent occurrence of such data points decreases the effective batch size, which often introduces noise. As training proceeds, weight distributions tend to narrowly center around values of 0 and 1, with decreasing number of values close to 0.5 (Supplementary Fig. 3). A suitable threshold for excluding insignificant samples is chosen depending on the weight distribution observed after training. Here, a weight threshold of 0.05 is applied in the first calculation setting and a threshold of 0.20 in the second setting (given the reduced number of pre- and meta-training epochs).

### Pre-training and fine-tuning

For each PK target $\:t$, two models are pre-trained on the source domains $\:{S}^{\left(-t\right)}$ containing a total number of 54,113 − 54,667 PK-PKI interactions (depending on the target). These models include the standard transfer learning model with uniform weights and the transfer learning model with weights obtained from meta-learning. The models are then fine-tuned on the data-reduced PK target using only 50 training PKIs obtained by stratified sampling from $\:{T}^{\left(t\right)}$ such that the overall class imbalance (active vs. inactive) is accounted for during training. Of note, test calculations were also carried out for 25 and 100 training PKIs. Sets of 100 training PKIs were already sufficiently large such that standard transfer learning had only marginal effects (Supplementary Fig. 4). On the other hand, using only 25 training PKIs resulted in a significant decrease in performance compared to the use of 50 PKIs, while similar trends were observed (Supplementary Fig. 5). The fine-tuned models are evaluated using the remaining PKIs for PK $\:t$.

Given the confined number of fine-tuning training compounds, statistically relevant differences in model performance might be observed in individual trials, depending on the composition of the fine-tuning sets. Therefore, a total of 50 independent fine-tuning trials were carried out for each target to ensure statistical robustness of the results. In each trial, fine-tuning test compounds were ranked according to the probability of activity produced by a model.

### Model evaluation

The compound rankings are evaluated using the receiver operating characteristic (ROC) curve^34^. The ROC curve compares the true positive and false positive rates across the ranking. The area under the ROC curve (AUC), ranging from 0 to 1, is calculated to quantify model performance. An AUC value of 0.5 indicates random classification and ranking whereas a value of 1 indicates a perfect classification (that is, all true positives are ranked higher than the first false positive). AUC value distributions are represented and compared using box plots. In addition, differences in AUC values between the meta- and control models are compared on a trial-by-trial basis using the non-parametric Wilcoxon signed-rank test to assess statistical significance.

To evaluate the effectiveness of the meta-learning approach in controlling negative transfer (see above), the mathematical definition of the negative transfer gap (NTG) by Wang et al.^15^ is applied. For a model $\:f$ trained only on the target domain and another model $\:h$ trained on the source domain and fine-tuned on the target domain, we define the negative transfer index (NTI) as follows:

8 $$\:NTI=\frac{1}{n}{\sum\:}_{i=1}^{N}\text{auc}\left(y,f\left(x;T\right)\right)-\frac{1}{n}{\sum\:}_{i=1}^{N}\text{auc}\left(y,h\left(x;T,S\right)\right)$$

where $\:N$ is the number of trials and $\:\text{auc}$ a function to estimate the AUC value. Accordingly, positive NTI values indicate the presence of negative transfer.

## Results

### Methodological concept

The major aim of our meta-learning approach is supporting transfer learning in low-data regimes. The basic idea is combining compound and target protein information to further refine weighting schemes for deep learning. By design, this approach is generally applicable to molecular property predictions such as activity-based compound classification. Therefore, models can be pre-trained in a source domain using weights for compound-target pairs determined by meta-learning. This provides an extended basis for fine-tuning using ligands of a new target protein. So-derived models are compared to standard models pre-trained and fine-tuned in the absence of meta-learning.

Specifically, the meta-learning method introduced herein identifies an optimal subset of source samples for pre-training of a base model. Fine-tuning is then performed on target data sets as in standard transfer learning. The meta-objective optimized during meta-training is the generalization performance of the pre-trained model on the target domain. Importantly, by operating at the instance level, negative transfer can be adaptively minimized for each target while increasing the generalization ability of the model. Compared to other meta-learning frameworks, the new algorithm has a number of unique features, is integrated with transfer learning, and strongly emphasis balancing of negative transfer. Therefore, direct comparisons with other available meta-learning approaches are not possible. For example, the major limitation of the MAML framework is its vulnerability to negative transfer. Therefore, we focus on providing proof-of-principle for the combined meta- and transfer learning approach and quantitative comparisons with standard transfer learning.

For proof-of-concept, we have chosen PKs and their inhibitors as a test system, for several reasons: PKs are closely related and for many PKs, large numbers of PKIs are available. Most currently available PKIs are directed against the ATP cofactor binding site in PKs that is largely conserved across the human kinome^35,36^. Accordingly, these ATP site-directed PKIs tend to be similar, represent a structural continuum, and are frequently active against more than one PK (multi-PK inhibitors)^36^. A subset of ~ 20–30% of currently available PKIs is known to be active against multiple PKs, giving rise to shared compounds in the source and target domains. Furthermore, for PKIs, practically relevant thresholds of activity can be applied to differentiate between related active (potent) and inactive (weakly potent) compounds. This presents challenges for PKI activity predictions compared to compound data sets where the negative (inactive) class is composed of randomly assembled (and thus irrelevant) compounds. Moreover, given the wealth of available PKs and PKIs, sufficient numbers of PKs can be converted into low-data targets for modeling by selecting small subsets of actives. Notably, PKI data have been used before to evaluate meta-learning with a framework closely related to MAML to predict inhibitors for low-data PKs^37^. For predicting PKIs, meta-learning was also combined with a graph-attention neural network learning a shared weight initialization for models covering a diverse set of PKs^38^.

### Models for data sets with shared source and target compounds

**Fig. 4:**
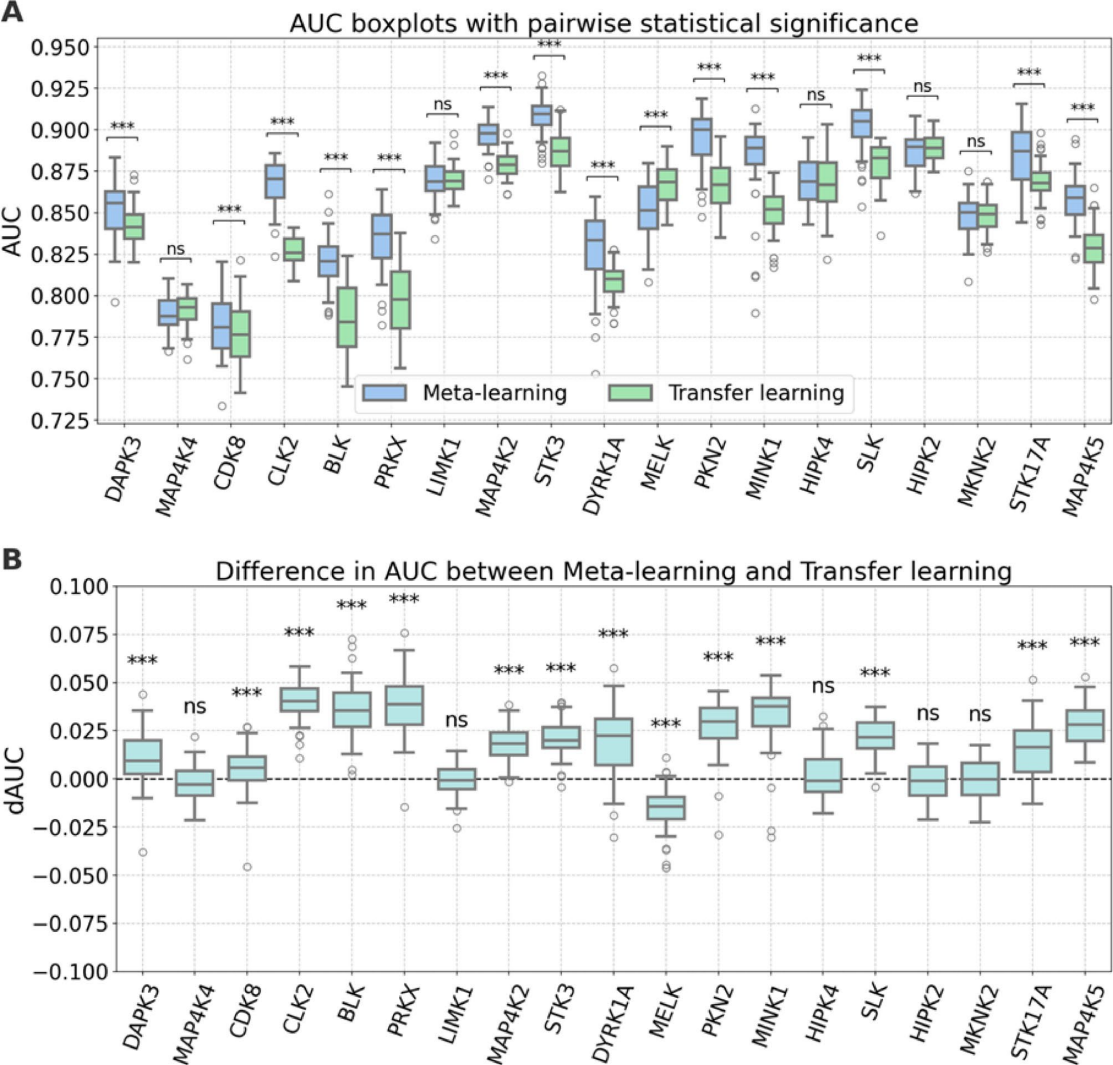
Model performance for the first calculation setting. (**A**) Shown are AUC boxplots (box: 1 st quartile, median, 3rd quartile; whiskers: +/− 1.0 x interquartile range; dots: outliers) for 50 independent trials of meta- and standard transfer learning models for the 19 target PKs. Statistical significance is indicated by asterisks; $\:0.05<\text{p-value}\le\:1$: ns (no statistical significance), $\:0.01<\text{p-value}\le\:0.05$: *, $\:0.001<\text{p-value}\le\:0.01$: **, and $\:\text{p-value}\le\:0.001$: ***. (**B**) Boxplots representing the distribution of differences in AUC (dAUC) on a per-trial basis are shown. Positive values indicate improved performance of meta-learning compared to standard transfer learning models.

We first investigated the effects of meta-learning when the source and target domains shared compounds. This was due to the presence of multi-PK inhibitors and generally favored standard transfer learning. For each of the 19 target PKs in Table 1, models were pre-trained in the presence and absence of weights from meta-learning, fine-tuned, and compared. In activity-based classification, median AUC values of the models ranged from ~ 0.75 to ~ 0.90, depending on the PK target, and independent trials often produced broad AUC value distributions (Fig. 4A). Hence, model performance was overall promising, but heterogeneous, leaving room for improvements. This was anticipated because designated active and inactive compounds represented a continuum of PKIs instead of discrete compound classes. However, in the presence of shared source and target compounds, meta-learning resulted in a statistically significant improvement for 13 of 19 PKs ($\:\text{p-value}<0.001$). By contrast, only one target (MELK) had a significant decrease in performance. The consistent improvements suggested that meta-learning had a further improved generalization capability compared to standard transfer learning. The absolute median increase in AUC as a consequence of meta-learning was generally small, ranging from 0.01 to 0.04 (Fig. 4A). However, this was partly a consequence of the broad AUC value distributions of independent trials. These distributions mirrored high variance when small PKI sets of different composition were used for fine-tuning. The consistency in improvements through meta-learning was much more evident when the difference in AUC (dAUC) was determined on a per-trial basis, as shown in Fig. 4B. For most of the PKs having a statistically significant difference, the boxes accounting for the interquartile range fell exclusively into the positive range, with only very few negative outliers.

#### Models for non-overlapping data sets of source and target compounds

**Fig. 5:**
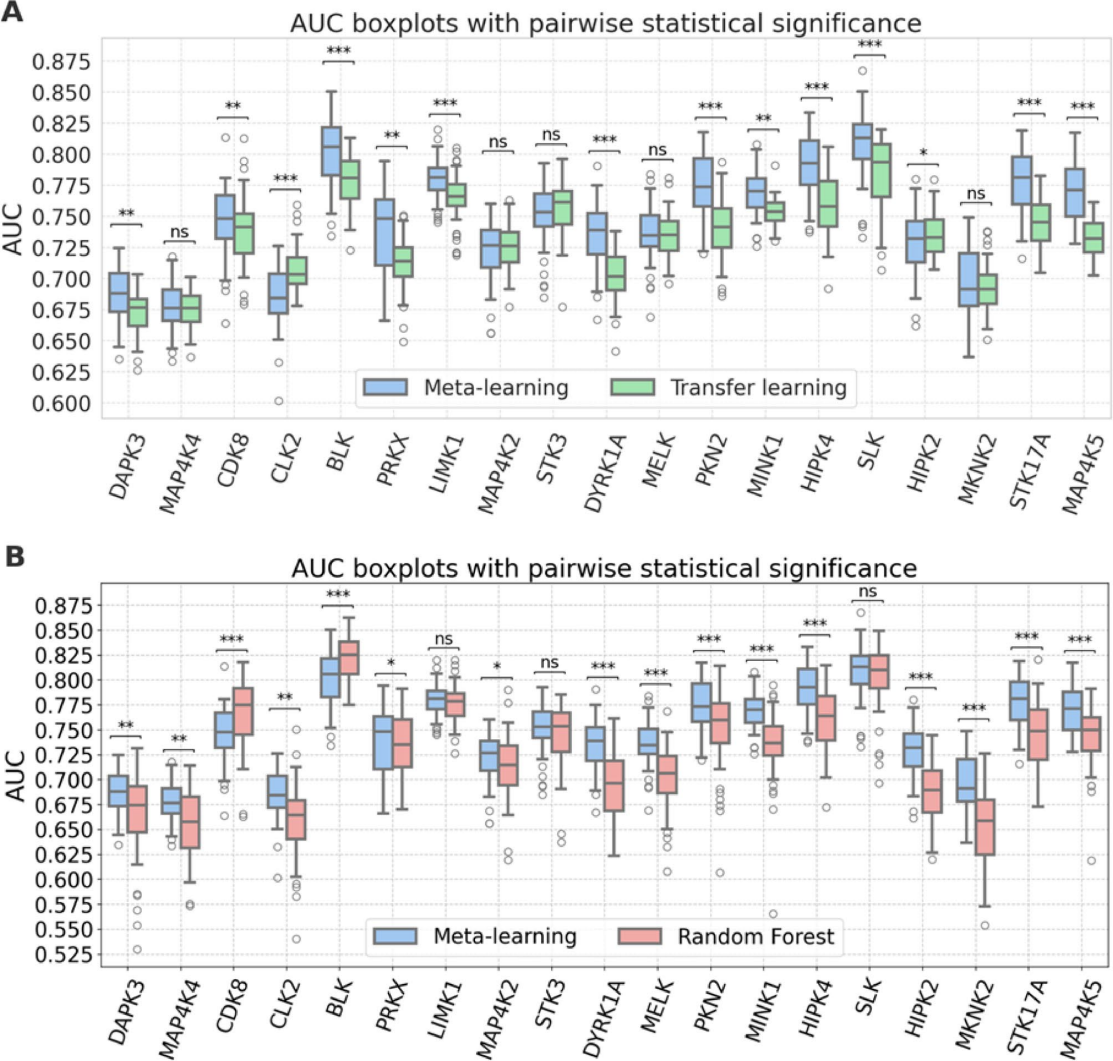
Model performance for the second calculation setting. (**A**) and (**B**) show AUC boxplots for 50 independent trials of meta-learning compared to standard transfer learning and RF models, respectively. Statistical significance of observed differences is reported according to Fig. 4.

**Fig. 6:**
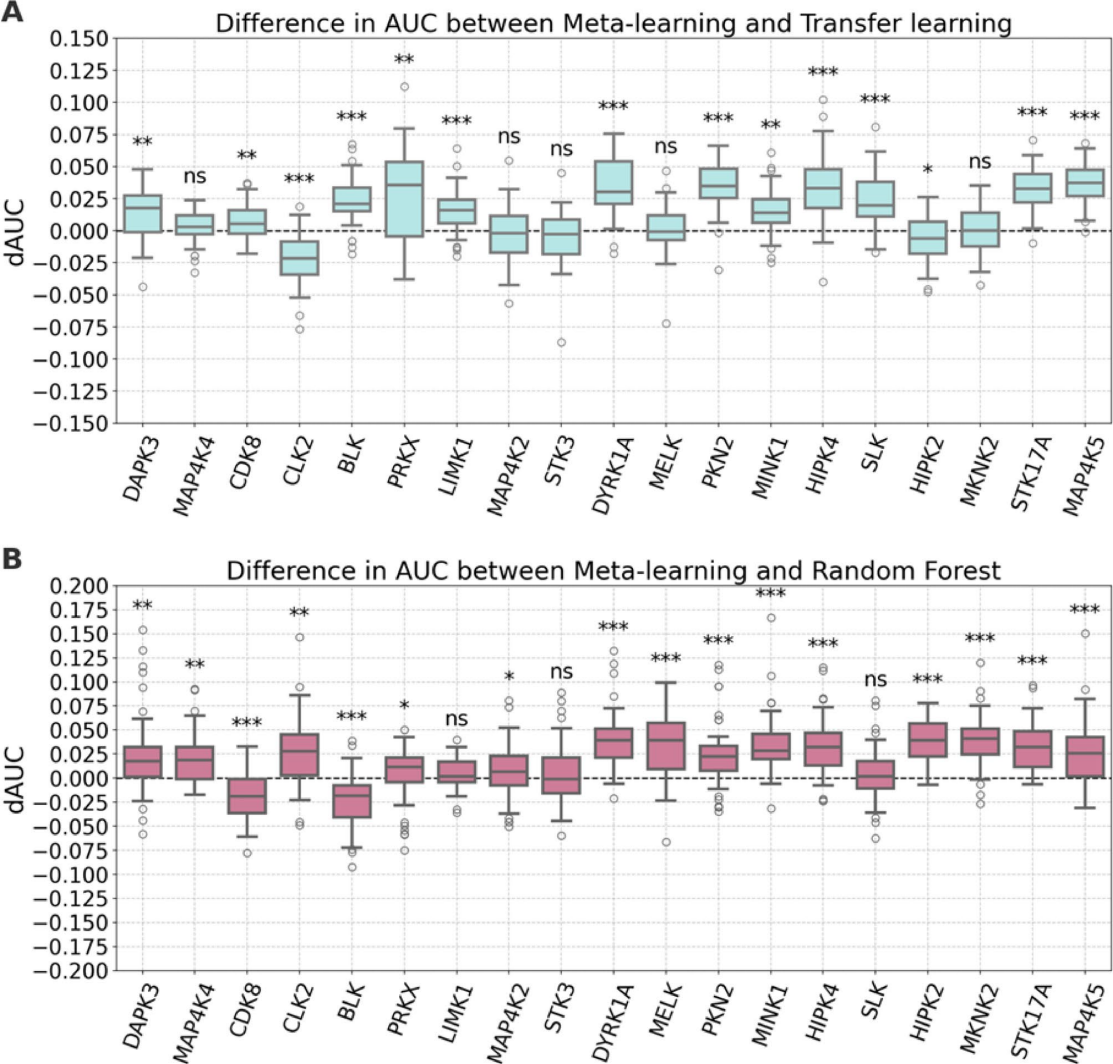
Performance differences on a per-trial basis for the second calculation setting. Boxplots show the distributions of dAUC values determined for each individual trial of meta-learning compared to (A) standard transfer learning and (B) RF models. Positive values indicate improved performance of meta-learning compared to other models. Statistical significance is reported according to Fig. 4.

Next, the models were re-generated after removal of shared source and target compounds (multi-PK inhibitors). Removal of shared compounds resulted in a significant reduction of the pre-training data $\:{S}^{\left(-t,-\cap\:\right)}$, ranging from 11,296 to 42,859 PK-PKI interactions, depending on the PK target. For non-overlapping compound sets, layer freezing strongly reduced the prediction accuracy of both deep learning models. Therefore, instead of retaining the learned patterns in a fixed bottleneck feature representation, fine-tuning was applied to all layers of the base model. Removal of shared compounds together with the ensuing reduction of training data further increased the difficulty of the prediction task. Therefore, we also included RF models in the analysis as a further reference. As anticipated, the performance of the re-generated models was reduced compared to the original models (Fig. 5). Median AUC values of the models ranged from ~ 0.65 to ~ 0.80, depending on the method and PK target. Compared to standard transfer learning, meta-learning yielded statistically significant improvements in performance for 12 of 19 PKs (Fig. 5A). Standard transfer learning produced a statistically significant improvement for only 1 PK (HIPK2). Similarly, compared to RF, meta-learning resulted in statistically significant improvements in performance for 14 PKs (Fig. 5B). RF models produced significantly improved performance in only two cases (CDK8, BLK). Thus, models built for non-overlapping source and target compound sets also yielded a preferential gain in performance as a consequence of meta-learning. As observed before for models built in the presence of shared source and target compounds, statistically significant gains in performance were generally of small magnitude, but consistent. This was further revealed by dAUC comparison of meta- and standard transfer learning on a pair-trial basis (Fig. 6A) as well as meta-learning and RF models (Fig. 6B).

### Negative transfer

**Fig. 7:**
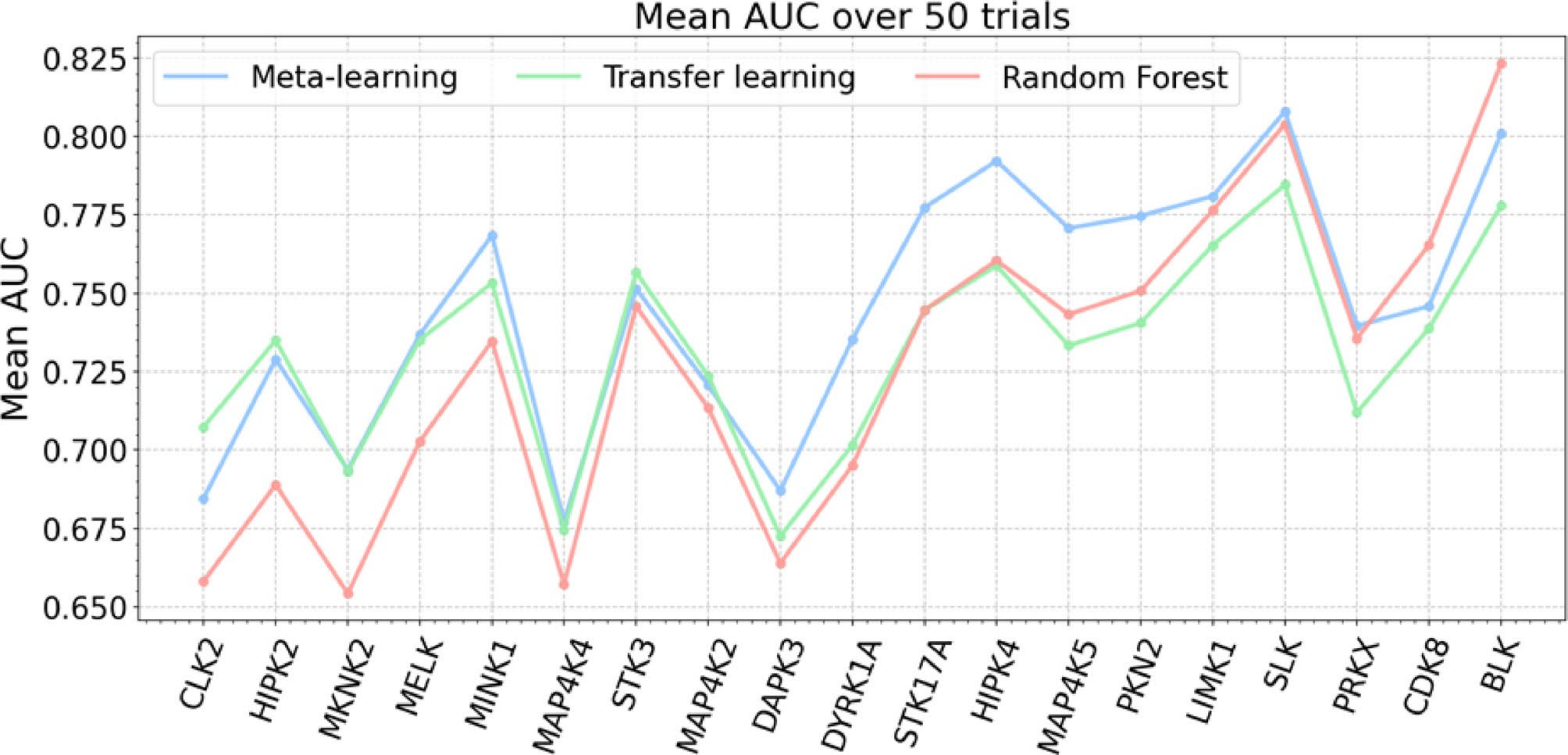
Assessment of negative transfer. For models based on non-overlapping source and target compound sets, mean AUC values over 50 independent trials are reported. The 19 PK targets are sorted in the order of ascending NTI values comparing RF and standard transfer learning models.

We then investigated the potential of the meta-learning method to control negative transfer. Therefore, NTI values were computed according to Eq. 8. Here, $\:f\left(x;T,{\zeta\:}^{}\right)$ represents the RF and $\:h\left(x;S,T,{\psi\:}^{}\right)$ the standard transfer learning model. In Fig. 7, target PKs were arranged in the order of ascending NTI values comparing RF and standard transfer learning models. Mean AUC values are reported for different models based on non-overlapping sets of source and target compounds. Thus, the tendency of negative transfer increased from the left to the right.

For the first PKs on the left, standard transfer learning resulted in a large AUC increase compared to the RF model, which indicated the presence of positive transfer. In this case, the performance of the meta-learning and standard transfer learning models was very similar. With beginning negative transfer, the performance of the standard transfer learning model became increasingly similar to the RF model while meta-learning yielded substantial performance increases. For PKs with largest negative transfer, the RF model met and then exceeded the performance of the meta-learning and standard transfer learning models. However, in these cases, meta-learning reduced negative transfer by ~ 50% compared to standard transfer learning. Thus, in the presence of increasing negative transfer, meta-learning achieved highest performance or at least balanced negative transfer compared to standard transfer learning. These findings indicated that the meta-learning algorithm effectively increased the relative weight of source samples that were relevant for the target tasks and reduced the weight of others that caused negative transfer.

## Conclusion

Machine learning in data-sparse domains generally benefits from the application of specialized learning concepts such as transfer or meta-learning, which rely on knowledge exchange between different domains. A pre-requisite for their application is the presence of related prediction tasks with varying amounts of available data to facilitate knowledge transfer to low-data tasks. Standard transfer learning typically relies on model pre-training in a source domain where sufficient data are available, followed by fine-tuning in the target domain with limited amounts of available data. On the other hand, meta-learning primarily aims at adapting models to new tasks without major training requirements, for instance, by learning of weight initializations. To this end, a distinguishing feature of meta-learning is the optimization of a meta-objective to ensure that models are readily adaptable.

We have reasoned that transfer and meta-learning are complementary in nature and might be integrated to further improve learning and prediction accuracy. Therefore, we have developed a new meta-learning algorithm that combines target protein (task) and compound (instance) information and acts as a front end of model pre-training for transfer learning. Accordingly, a model is pre-trained in a source domain using weights for compound-target interactions determined by the meta-learning model. This provides an advanced basis of fine-tuning in a target domain. The meta-objective of the model is optimization of the generalization potential of the pre-trained model in the target domain. Therefore, the meta-learning algorithm identifies the most suitable subset of compounds for pre-training.

In our proof-of concept application, meta-learning led to statistically significant improvements in prediction accuracy for the majority of tasks. For low-data applications, these improvements are relevant, given their consistency. Moreover, since the meta-learning algorithm also works at the instance level (leading to the identification of optimized training compound subsets), it is applicable to explicitly address the problem of negative transfer, a key limitation of standard transfer learning. We demonstrated that the use of the meta-model as a transfer learning front end effectively controlled negative transfer effects during the predictions. Therefore, the consistent improvements in prediction accuracy should also be considered in the context of balanced negative transfer, rendering the new approach more robust than standard transfer learning for any applications affected by negative transfer. Taken together, our findings indicate that the meta-learning algorithm introduced herein provides new opportunities for machine learning in low-data regimes. Although the methodology was primarily conceived for cheminformatics, it is generally applicable and can be adopted in other fields.

## Supplementary Information

Below is the link to the electronic supplementary material.

Supplementary Material 1

## Data Availability

Data and code generated for this study are available via the following link: https://uni-bonn.sciebo.de/s/LRP43AmLaWA5Xc6.
