# Supplementary material for: A meta-learning framework to mitigate negative transfer in transfer learning applicable to drug design
Source: Sci Rep. 2025 Oct 9;15:35236. doi: 10.1038/s41598-025-22058-3 (PMC12511290; doi:10.1038/s41598-025-22058-3)
Supplement: Supplementary file 1 — Supplementary Material 1 [file 41598_2025_22058_MOESM1_ESM.docx]

**Supplementary Material**

**A meta-learning framework to mitigate negative transfer in transfer learning applicable to drug design**

Antonia Mera,^1,2^ Martin Vogt,^1,2^ and Jürgen Bajorath^1,2^*

^1^Department of Life Science Informatics and Data Science, B-IT, LIMES Program Unit Chemical Biology and Medicinal Chemistry,

^2^Lamarr Institute for Machine Learning and Artificial Intelligence, University of Bonn

Friedrich-Hirzebruch-Allee 5/6, D-53115 Bonn, Germany

*Correspondence:

Tel: +49-228-7369-100, Fax: +49-228-7369-100, E-mail: bajorath@bit.uni-bonn.de

| **A**  **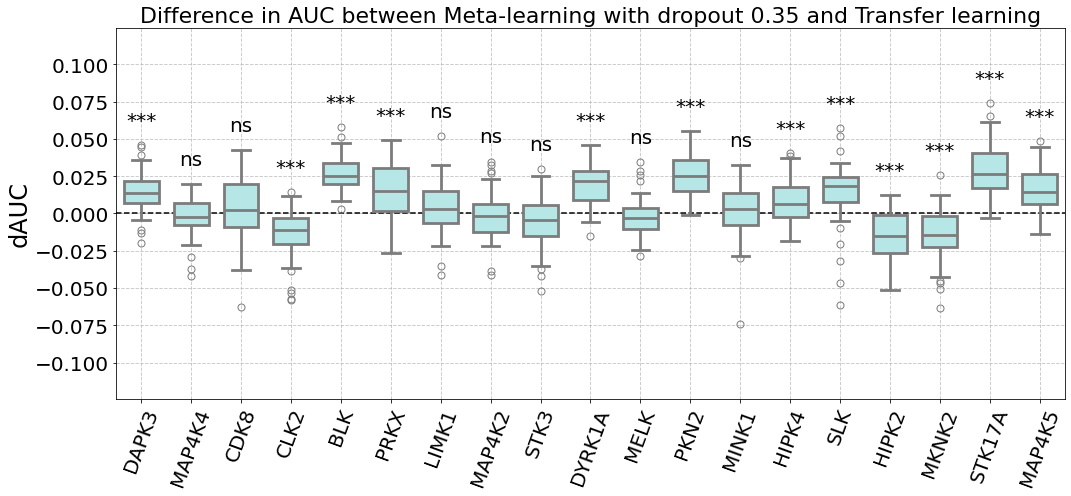** |
| --- |
| **B**  **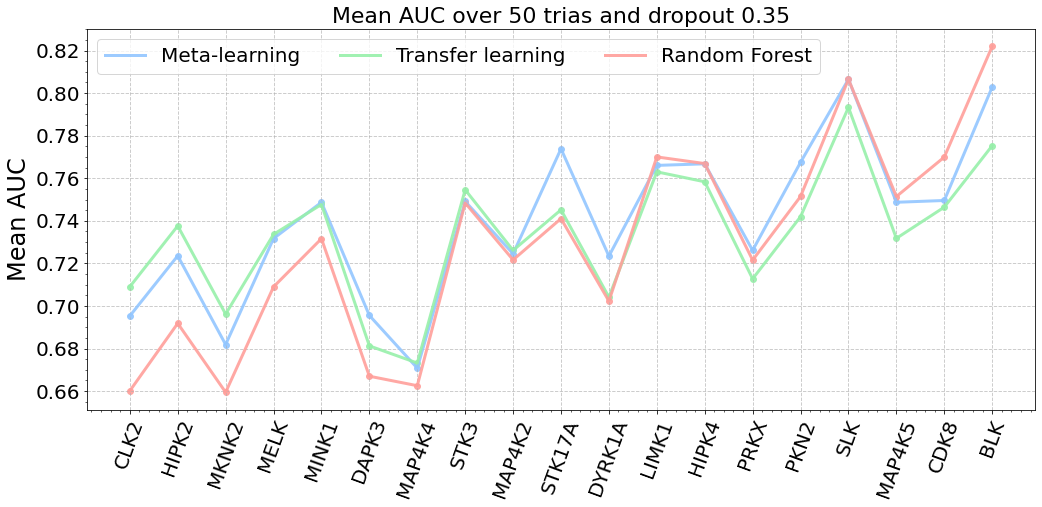** |

**Supplementary Figure 1.** Performance differences and assessment of negative transfer with dropout rate variation. For the second calculation setting (non-overlapping data sets, corresponding to Figure 6A and 7), results are reported for a dropout rate of 0.35. (A) Boxplots show the distributions of dAUC values determined for each individual trial for meta-learning compared to transfer learning. (B) For models based on non-overlapping source and target compound sets, mean AUC values over 50 independent trials are reported. The 19 PK targets are given in order of ascending NTI values, comparing RF and transfer learning models.

| **A**  **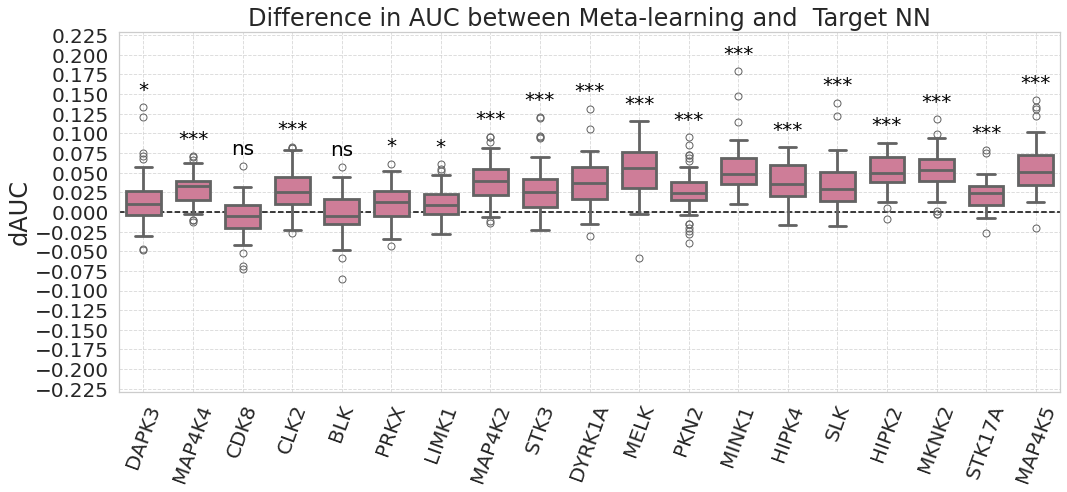** |
| --- |
| **B 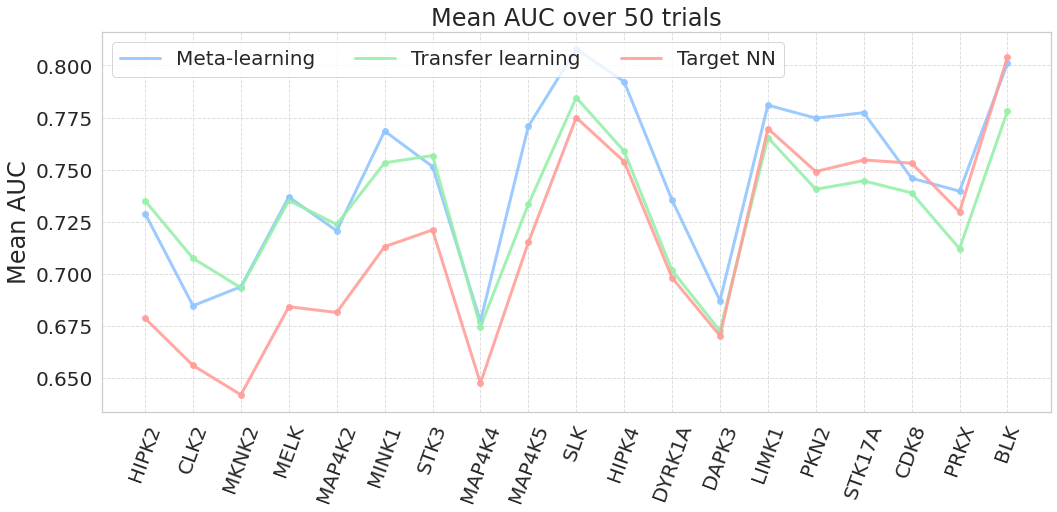** |

**Supplementary Figure 2.** Performance differences and assessment of negative transfer for the neural network control model. Results are reported for a sample size of 50. (A) Boxplots show the distributions of dAUC values determined for each individual trial for meta-learning compared to a neural network control model trained only on the target data set. (B) For models based on non-overlapping source and target compound sets, mean AUC values over 50 independent trials are reported. For the 19 NTI-ordered PK targets, the base model trained only on the target domain and transfer learning models are compared.

| **A**  **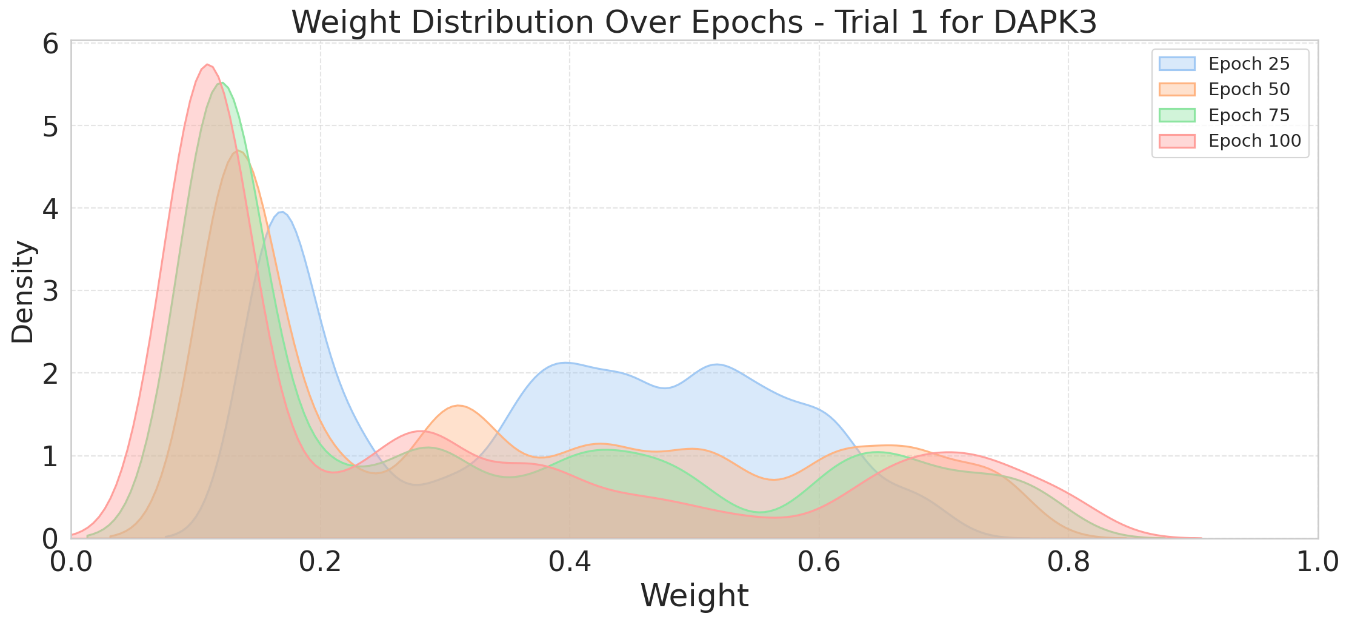** |
| --- |
| **B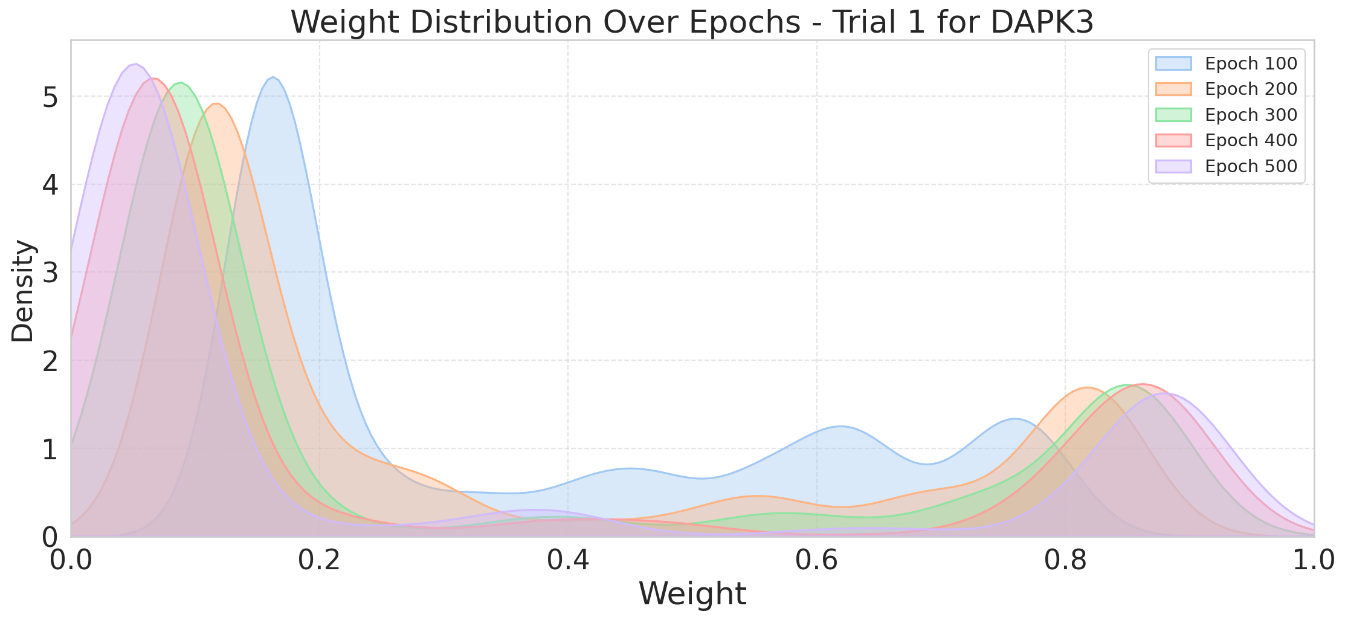** |

**Supplementary Figure 3.** Weight distributions. Shown are weight distributions of samples from the source domain, as predicted by meta-learning for the DAPK3 target at different epochs. For other PKs, similar trends are observed for the predicted weight distributions. (A) Weight distributions for epochs 25 to 100. (B) Weight distributions for epochs 100 to 500.

| **A**  **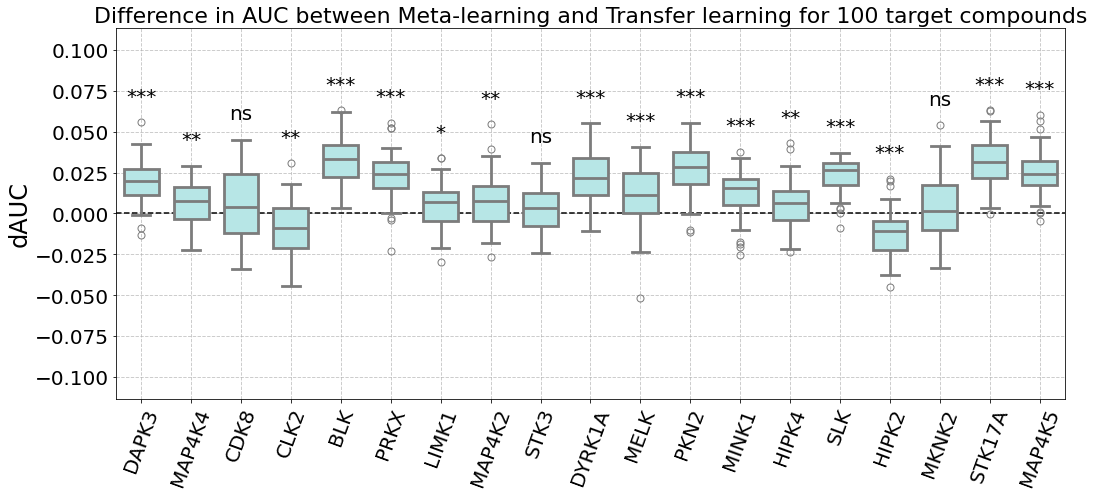** |
| --- |
| **B**  **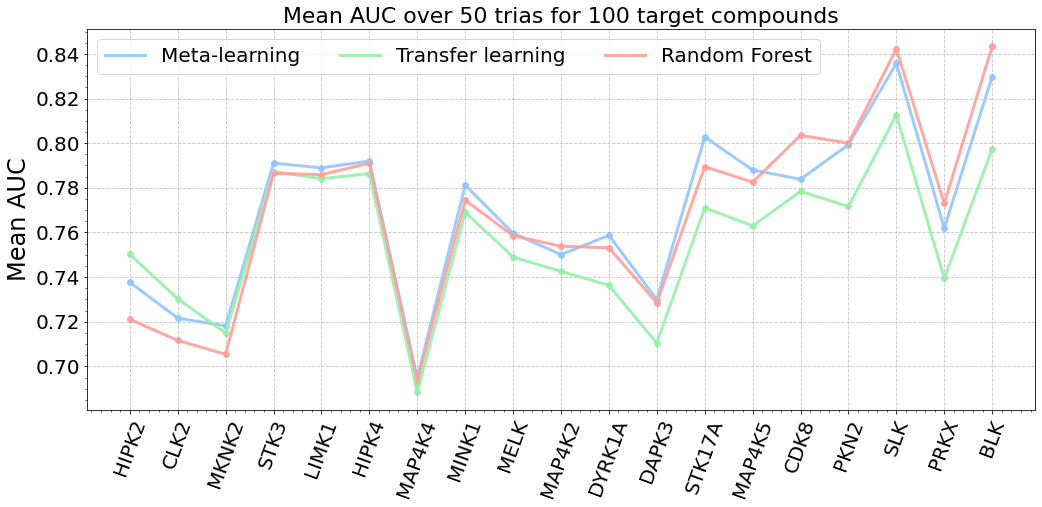** |

**Supplementary Figure 4.** Performance differences and assessment of negative transfer with sample size variation. For the second calculation setting, results are reported for a target sample size of 100. (A) Boxplots show the distributions of dAUC values determined for each individual trial for meta-learning compared to transfer learning. (B) For models based on non-overlapping source and target compound sets, mean AUC values over 50 independent trials are reported. The 19 target PKs are given in order of ascending NTI values, comparing RF and transfer learning models.

| **A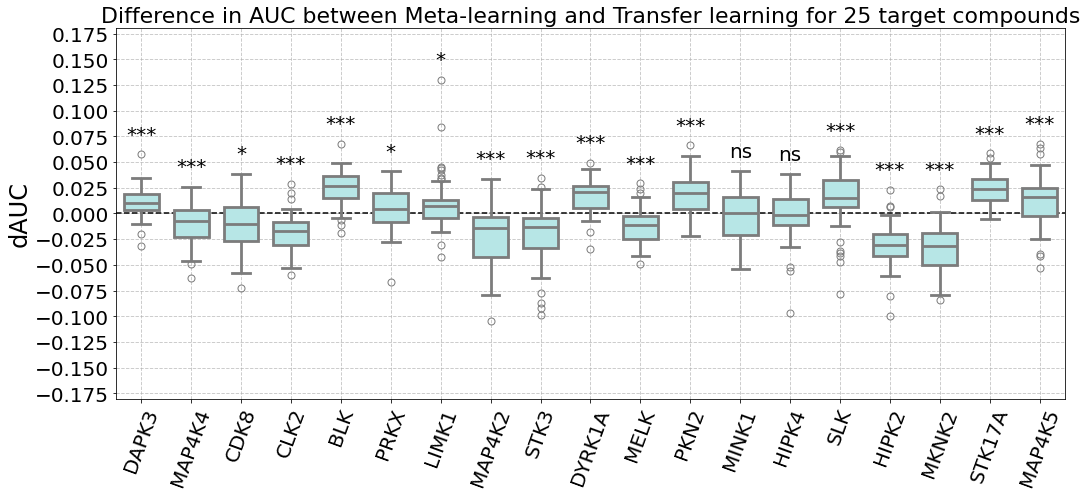** |
| --- |
| **B** 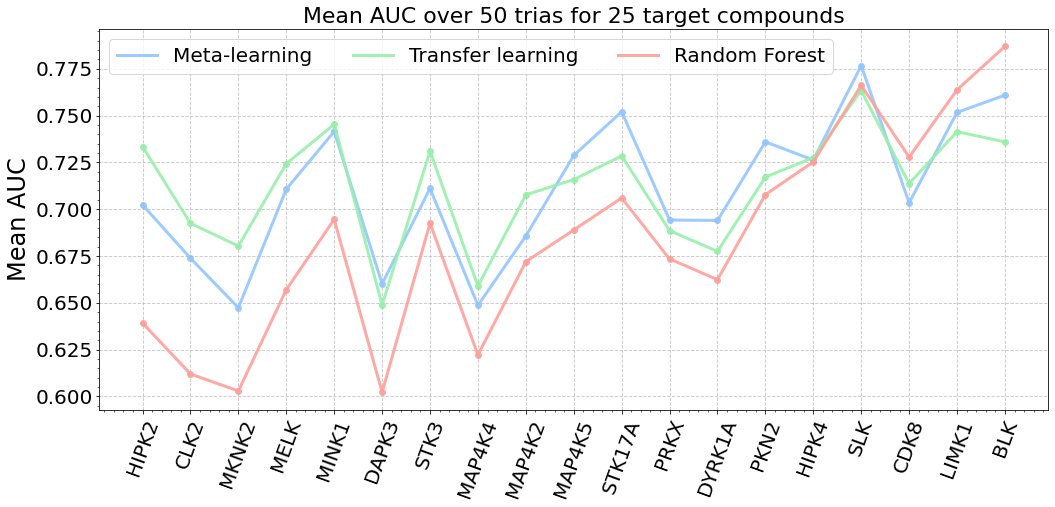 |

**Supplementary Figure 5.** Performance differences and assessment of negative transfer with sample size variation. For the second calculation setting, results are reports for a target sample size of 25. (A) Boxplots show the distributions of dAUC values determined for each individual trial for meta-learning compared to transfer learning. (B) For models based on non-overlapping source and target compound sets, mean AUC values over 50 independent trials are reported. The 19 target PKs are given in order of ascending NTI values, comparing RF and transfer learning models.
